# Applicability and Psychometric Comparison of the General-Population Viral Anxiety Rating Scales among Healthcare Workers in the COVID-19 Pandemic

**DOI:** 10.3390/ijerph19169946

**Published:** 2022-08-12

**Authors:** Changnam Kim, Oli Ahmed, Washington Allysson Dantas Silva, C. Hyung Keun Park, Soyoung Yoo, Seockhoon Chung

**Affiliations:** 1 Department of Psychiatry, Samsung Changwon Hospital, Sungkyunkwan University of Medicine, Changwon 06351, Korea; 2 Department of Psychology, University of Chittagong, Chattogram 4331, Bangladesh; 3 National Center for Epidemiology and Population Health, Australian National University, Canberra 2601, Australia; 4 Department of Psychology, Universidade Federal da Paraíba, João Pessoa 58051-900, PB, Brazil; 5 Department of Psychiatry, Asan Medical Center, University of Ulsan College of Medicine, Seoul 05505, Korea; 6 Department of Convergence Medicine, Asan Medical Center, University of Ulsan College of Medicine, Seoul 05505, Korea

**Keywords:** COVID-19, anxiety, stress, validation, SAVE-9

## Abstract

We aimed to explore the reliability and validity of viral anxiety rating scales (developed for the general population) among healthcare workers. In addition, we compared the psychometric properties of rating scales in accordance with the Generalized Anxiety Scale-7 items (GAD-7) during this COVID-19 pandemic. The viral anxiety of 330 healthcare workers was measured with Stress and Anxiety to Viral Epidemics—9 items (SAVE-9), SAVE-6, Coronavirus Anxiety Scale (CAS), Fear of COVID-19 Scale (FCV-19S), and COVID-19 Anxiety Scale (CAS-7). Factor analyses, item response theory, and Rasch model analyses were conducted to confirm the construct validities of the scales and compare the psychometric properties of rating scales. The receiver operating curve (ROC) analysis examined the cutoff scores of rating scales in accordance with a mild degree of generalized anxiety. The SAVE-9, SAVE-6, CAS, FCV-19S, and CAS-7 scales showed good reliability of internal consistency among healthcare workers. Their construct validity and convergent validity of each scale were similarly good. Furthermore, in comparing the psychometric properties of rating scales, we observed that the CAS scale was the most discriminating and difficult among the scales. The CAS and FCV-19S provided more information and were more efficient than the SAVE-9, SAVE-6, and CAS-7 scales when they were used to measure healthcare workers’ viral anxiety. Viral anxiety rating scales can be applied to healthcare workers with good reliability and validity.

## 1. Introduction

Until the early 2000s, with the development of vaccines and communicable diseases control, concerns about viral diseases decreased [1]. However, new viral infections such as the Ebola virus epidemic in 2014 and the MERS virus epidemic in 2015 [2] started to appear. In December 2019, an outbreak of COVID-19 in Wuhan, China, swept the world, with 572,239,451 confirmed cases and 6,390,401 deaths recorded as of 29 July 2022 [3]. To worsen matters, in May 2022, Monkeypox (Monkeypox virus), a disease endemic to Africa emerged in Europe and is unusually also present in countries that are not endemic to the disease. With the development of transportation, the rate of spread of infectious diseases is also increasing; thus, it can be assumed that a high possibility exists of other viral diseases occurring worldwide in the future.

As a result of the COVID-19 pandemic, people’s lives and physical health have been seriously endangered. Additionally, it has triggered panic disorders, anxiety, and depression. High prevalence of depression, anxiety, or post-traumatic stress symptoms has been reported among the general population in various countries [4,5,6,7,8,9,10] In addition, stress and anxiety among various population such as schoolteachers, firefighters, or public workers has also been reported [11,12,13].

The mental health of healthcare workers has also been threatened in various ways [1]. Maintaining quarantine rules at the front line and treating more patients than usual due to COVID-19 with limited medical resources led to an overall increase in workload and work intensity [14]. As the pandemic continues, the situation requiring the sacrifice of healthcare workers is protracted, which results in increased stress and burnout of healthcare workers [15]. The anxiety felt by healthcare workers at the treatment scene also acts as an important factor threatening the mental health of healthcare workers [1]. Previous studies found that the continuing COVID-19 pandemic causes various mental problems such as depression, anxiety, insomnia, and post-traumatic stress in the general population [16]. Even if quarantine rules are strictly followed, most healthcare workers are repeatedly exposed to the risk of infection as they are continuously exposed to highly contagious viruses while taking care of COVID-19-infected patients. Continuous exposure to novel viral diseases that are rapidly transmitted and not fully explored is likely to cause anxiety among healthcare workers about infection [17,18].

During this COVID-19 pandemic, frontline healthcare workers suffer from psychological distress when they are working and taking care of infected patients. Psychological support systems for healthcare workers are needed for themselves and the patients whom they take care of. In addition, healthcare worker-specific and viral epidemic-specific rating scales are needed. Several rating scales such as the Coronavirus Anxiety Scale (CAS) [19], Fear of COVID-19 Scale (FCV-19S) [20], COVID-19 Anxiety Scale [21], or Stress and Anxiety to Viral Epidemics—6 items Scale (SAVE-6) [22] were developed for the general population. However, only one rating scale, the Stress and Anxiety to Viral Epidemics—9 items Scale (SAVE-9) [23], was developed for healthcare workers. The SAVE-9 was developed to measure work-related stress and anxiety response specific to viral epidemics, while the SAVE-6 was derived from the SAVE-9 scale to apply to the general population. Although we explored the applicability of the SAVE-6 scale in a sample of healthcare workers [24], we could not compare the psychometric properties between the SAVE-9 and SAVE-6 scales among healthcare workers. Since each rating scale has clinical characteristics to be considered when these are applied to individuals, we need various kinds of rating scales that can be applied to healthcare workers with good reliability and validity.

In this study, we aimed to explore the reliability of internal consistency and construct validity of rating scales, including the SAVE-9, SAVE-6, FCV-19S, CAS-7, and CAS, as a single factor among a sample of healthcare workers. In addition, we tried to compare the psychometric properties and convergent validity of these rating scales in accordance with the Generalized Anxiety Scale-7 (GAD-7) items among healthcare workers in this pandemic.

## 2. Materials and Methods

### 2.1. Study Design

This survey was conducted online on 29 November 2021, among healthcare workers in Asan Medical Center, University of Ulsan College of Medicine, Seoul, Korea. The participants voluntarily completed the survey, and we provided a gift coupon valued at about 5 USD to the participants. The study protocol was approved by the Institutional Review Board (IRB) of the Asan Medical Center (2021-1682), and the need to obtain written informed consent was waived by the IRB.

### 2.2. Sampling Procedure

Healthcare workers of Asan Medical Center were recruited via an advertisement posted on hospital’s intranet which employers logged into every day. A survey form was developed using Google Forms^®^ (Google LLC, Mountain View, CA, USA), and the link to access the survey was posted on the intranet. We informed participants that personal information such as name, work unit, or any identifiable information would not be collected. Via the online survey, participants’ information on age, job (medical doctors, nursing professionals, or other) sex, years of employment, and marital status were collected. Participants responded to questions on COVID-19 such as being quarantined, being infected, or getting vaccinated. Their past psychiatric history and current psychiatric distress were asked. The e-survey form was developed according to the Checklist for Reporting Results of Internet e-Surveys (CHERRIES) guidelines [25], and the investigator (S.C.) tested the usability and technical functionality of the e-survey before implementation.

The sample size was estimated as 320 on the basis of the allocation of 40 samples for 10 cells; two groups of biological sex (male and female) × four age groups (18–29, 30–39, 40–49, and 50–65 years old [26]. In addition, the sample size required to determine whether a correlation coefficient differs from zero was 347 (type I error (α) = 0.05, type II error (β) = 0.20, and expected correlation coefficient = 0.15). We decided to collect 330 samples among 9216 workers (1759 medical doctors, 4526 nursing professionals, and 2931 other workers) in the hospital. All required responses were collected in 1 day; therefore, no further responses were collected.

### 2.3. Measures

#### 2.3.1. Stress and Anxiety to Viral Epidemics—9 Items and —6 Items (SAVE-9 and SAVE-6)

The SAVE-9 scale was developed to measure healthcare workers’ work-related stress and anxiety response specifically to the viral epidemic [23]. It consists of nine items which can be rated on a five-point Likert scale from 0 (never) to 4 (always). A higher total score, ranging from 0 to 36, reflects levels of work-stress and viral anxiety. The SAVE-9 scale was clustered into two factors: factor I—anxiety about the epidemic (items 1, 2, 3, 4, 5, and 8) and factor II—work-related stress associated with the epidemic (items 6, 7, and 9). The SAVE-6 was derived from factor I of the SAVE-9 scale to measure viral anxiety of the general population [22]. In this study, we used the original Korean version of the scales.

#### 2.3.2. Coronavirus Anxiety Scale (CAS)

The CAS is a brief self-reported rating scale to screen clinical anxiety and fear related to the COVID-19 crisis or “corona phobia” [19]. All five items of the CAS can be rated on a five-point Likert scale from 0 (not at all) to 4 (nearly every day). A higher total score, ranging from 0 to 20, reflects higher levels of fear of COVID-19. We applied the Korean version of CAS [27] in this study.

#### 2.3.3. Fear of COVID-19 Scale (FCV-19S)

The FCV-19S is a self-reported rating scale to measure one’s viral anxiety [20]. All seven items of the FCV-19S can be rated on a five-point Likert scale from 1 (strongly disagree) to 5 (strongly agree). A higher total score, ranging from 7 to 35, reflects higher levels of anxiety toward viral diseases. We applied the Korean version of FCV-19S in this study [28].

#### 2.3.4. COVID-19 Anxiety Scale (CAS-7)

The CAS-7 scale is a self-reported rating scale which can measure one’s anxiety toward viral diseases [21]. All seven items can be rated on a four-point Likert scale from 0 (does not apply to me) to 3 (applies to me). The higher total score, ranging from 0 to 21, reflects greater anxiety regarding COVID-19. We translated the CAS-7 scale into the Korean version using translation and back-translation methods with the permission of the developer. A bilingual expert translated the original scale into the Korean version, and the Korean version was back-translated into English to check accuracy.

#### 2.3.5. Generalized Anxiety Disorder-7 Items (GAD-7)

The GAD-7 is a self-report rating scale which can measure one’s general anxiety [29]. All seven items can be rated on a four-point Likert scale from 0 (not at all) to 3 (nearly every day). A higher total score, ranging from 0 to 21, reflects a severe degree of general anxiety. In this study, we applied the Korean version of the GAD-7 scale [30].

### 2.4. Statistical Analysis

We explored the reliability of internal consistency and construct or convergent validity of rating scales. In the first step, we examined the construct validity of the single factor structure of each scale using confirmatory factor analysis (CFA) among healthcare workers. CFA with diagonally weighted least squares (DWLS) methods was conducted, and satisfactory model fit was defined by a standardized root-mean-square residual (SRMR) value ≤0.05, root-mean-square-error of approximation (RMSEA) value ≤0.10, and comparative fit index (CFI) and Tucker–Lewis index (TLI) values ≥0.90 [31,32]. Multi-group CFA was conducted to examine whether each scale could measure the viral anxiety in a same way across sex or having depression. Reliability of internal consistency was examined using Cronbach’s α and McDonald’s ω. Psychometric properties were also assessed by conducting the item response theory (IRT) approach (graded response model (GRM)) and Rasch analysis. In GRM, item fits (assessed through S-χ^2^ and its *p*-values (adjusted for false discovery rate)) were assessed for each scale. There are two parameters in GRM—slope/discrimination parameter (α) and threshold/difficulty parameters (b) of items. Both parameters in GRM were estimated using the R package version mirt version 1.34. IRT reliability was also calculated. In the Rasch analysis, weighted fit statistics (iInfit) and outlier sensitive fit statistic (outfit) mean square (MnSq) were used at the item level, while item and person separation reliability, and item and person separation index were applied at the scale level. MnSq values close to 1 suggest good model–data fit. The accepted range of infit MnSq and outfit MnSq values is between 0.5 and 1.5 [33]. Recommended item and person reliability values are 0.7 or larger [34], and recommended item and person separation indices are 2 or larger [35].

In the second step, we compared psychometric properties of the SAVE-6, SAVE-9, CAS, FCV-19, CAS-7, and GAD-7 scales among healthcare workers. Convergent validity with GAD-7 was examined using Pearson’s correlation analysis. Receiver operating curve (ROC) analysis was conducted to explore and compare the cutoffs of each rating scale according to the mild degree (5 points) of the GAD-7 scale. The SPSS version 21.0, AMOS version 27 (SPSS, Inc, Chicago, IL, USA), JASP version 0.14.1.0 software (JASP Team, Amsterdam, The Netherlands), Rasch analysis, and DIF were run through jMetrik version 4.1.1 software (J Patrick Meyer, Charlottesvillle, VA, USA), and RStudio was used for statistical analysis.

## 3. Results

All 330 healthcare workers participated in this survey, and 329 agreed to the use of their responses for the study purposes. Among 329 participants, 264 (81.4%) were female and 194 (59.0%) were nursing professionals, 45 (13.7%) experienced being quarantined, two (0.6%) experienced being infected, and 327 (99.4%) were vaccinated (Table 1).

### 3.1. Reliability and Validity of the SAVE-9, SAVE-6, CAS, FCV-19S, and CAS-7 Scales among Healthcare Workers

The CFA with DWLS showed a good model fit for the SAVE-9 (CFI = 0.999, TLI = 0.999, RMSEA = 0.011, and SRMR = 0.049), SAVE-6 (CFI = 1.000, TLI = 1.000, RMSEA = 0.000, and SRMR = 0.034), CAS (CFI = 1.000, TLI = 1.114, RMSEA = 0.000 and SRMR = 0.059), FCV-19S (CFI = 1.000, TLI = 1.000, RMSEA = 0.005, and SRMR = 0.041), and CAS-7 (CFI = 0.974, TLI = 0.960, RMSEA = 0.084, and SRMR = 0.089) scales (Table 2). Factor loadings of each scale are shown in Table 3. The multigroup CFA revealed that all scales assessed viral anxiety in the same way across sex and having depression.

Good reliability of internal consistency of all viral anxiety rating scales was shown among healthcare workers (Table 2). All the scales had acceptable to good IRT reliability (0.633–0.881) and rho coefficient (0.800–0.897). Convergent validity was observed by Pearson’s correlation, and we observed that the SAVE-9 scale was significantly correlated with the SAVE-6 (*r* = 0.937, *p* < 0.001), CAS (*r* = 0.384, *p* < 0.001), FCV-19S (*r* = 0.615, *p* < 0.001), CAS-7 (*r* = 0.652, *p* < 0.001), and GAD-7 (*r* = 0.355, *p* < 0.001). The SAVE-6 scale was correlated with CAS (*r* = 0.342, *p* < 0.001), FCV-19S (*r* = 0.617, *p* < 0.001), CAS-7 (*r* = 0.642, *p* < 0.001), and GAD-7 (*r* = 0.289, *p* < 0.001). The CAS was correlated with FCV-19S (*r* = 0.484, *p* < 0.001), CAS-7 (*r* = 0.512, *p* < 0.001), and GAD-7 (*r* = 390, *p* < 0.001). The FCV-19S was correlated with the CAS-7 (*r* = 0.782, *p* < 0.001) and GAD-7 (*r* = 352, *p* < 0.001). The CAS-7 scale was correlated with GAD-7 (*r* = 442, *p* < 0.001).

### 3.2. Graded Response Model

Table 4 shows the GRM outputs of the scales. For SAVE-9, slope/discrimination parameters (α) ranged between 0.700 and 2.597 (mean = 1.472). Items 5, 6, 7, and 9 had moderate slope parameters, items 1 and 8 had high slope parameters, and the remaining items had very high slope parameters. Regarding the threshold parameters, items 5 and 6 were more difficult compared to the rest of the items. A higher latent trait or theta was required to endorse response option “sometimes” to “always” in items 5 and 6. For the remaining items, a higher latent trait was required to endorse response option “always”. For SAVE-6, slope/discrimination parameters (α) ranged between 1.099 and 2.778 (mean = 1.788). Items 5 and 6 had moderate slope parameters, item 1 had a high slope parameter, and the remaining items had very high slope parameters. Regarding the threshold parameters, item 5 was more difficult than the remaining items as a higher latent trait or theta was required to endorse the response option “sometimes” to” “always” in this item. For the remaining items, a higher latent trait was required to endorse response option “always”. 

**Figure 1:**
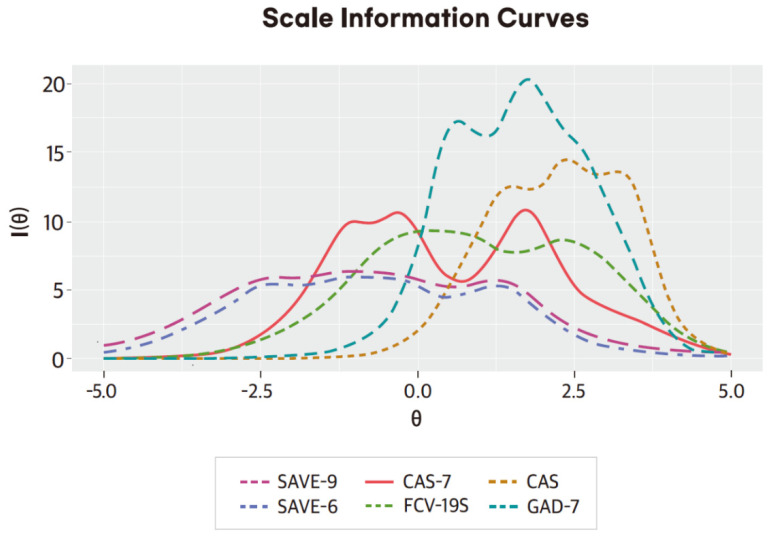
Scale information curves of rating scales.

For CAS, slope/discrimination parameters (α) ranged between 2.314 and 4.158 (mean = 3.168, Table 4). All the items had very high slope parameters, suggesting greater efficiency to provide information about the latent trait. Threshold parameters showed that a higher latent trait or theta was required to endorse the very first response option in all items. For FCV-19, slope/discrimination parameters (α) are ranged between 1.695 and 2.828 (mean = 2.180) (Table 4). All items had very high slope parameters, suggesting greater efficiency to provide information about the latent trait. Threshold parameters showed that, among the items, item 2 was the least difficult and item 3 was the most difficult. For CAS-7, slope/discrimination parameters (α) ranged between 1.826 and 3.930 (mean = 2.427). All items had very high slope parameters, suggesting greater efficiency to provide information about the latent trait. Threshold parameters showed that items 1, 3, and 7 were the least difficult items, and item 6 was the most difficult item. For GAD-7, slope/discrimination parameters (α) ranged between 1.791 and 4.745 (mean = 3.359). All items had very high slope parameters, suggesting greater efficiency to provide information about the latent trait. Threshold parameters showed that all the items were relatively difficult items. Scale information curves of all the scales (Figure 1) showed that both SAVE-6 and SAVE-9 provided almost the same level of information, but CAS, FCV-19, CAS-7, and GAD-7 provided more information than these two scales. There were several peaks in curves that may have been the result of Likert-type data.

### 3.3. Rasch Analysis

Item fit statistics (Table 5) showed that each item of all the scales met the unidimensional requirement of a Rasch model as all the values were within the 0.5–1.5 range. For both SAVE-9 and SAVE-6, item 1 was the least difficult and item 5 was the most difficult. For CAS, item 2 was the least difficult and item 3 was the most difficult. For FCV-19, item 2 was the least difficult and item 6 was the most difficult. For CAS-7, item 7 was the least difficult and item 6 was the most difficult. For GAD-7, item 3 was the least difficult and item 5 was the most difficult. Table 5 also shows the item and person reliability and separation index values of all the scales. Although all scales had acceptable item reliability and separation indices, CAS failed to meet the cutoff scores for the person reliability and separation index.

### 3.4. Comparison of the Psychometric Properties of the SAVE-9 with Other Viral Anxiety Rating Scales among Healthcare Workers

**Figure 2:**
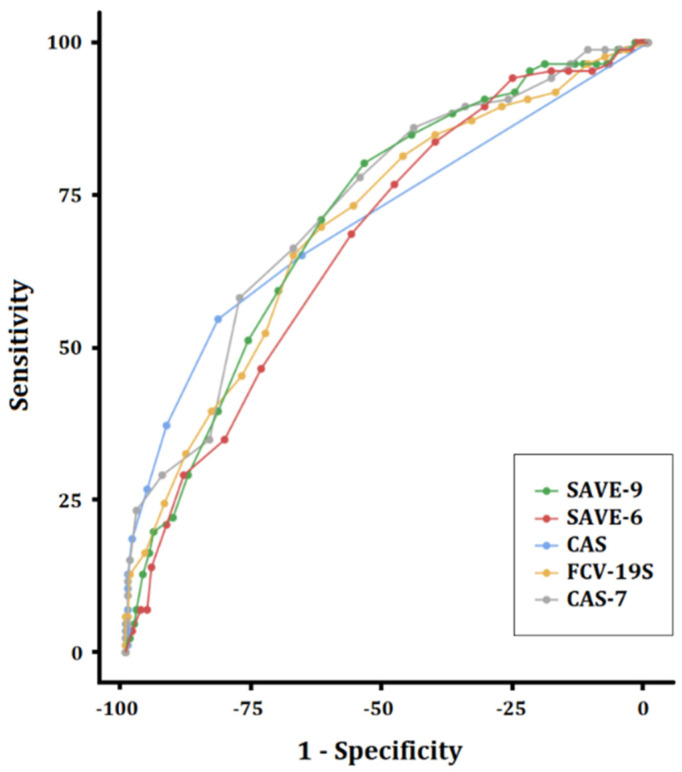
Receiver operating curve for viral anxiety rating scales according to five points of the Generalized Anxiety Disorder—7 items scale.

From the ROC analysis, which was conducted to explore the cutoff score of each rating scale according to five points of the GAD-7 scale (mild degree), the area under the curve (AUC) of each scale was 0.711 for SAVE-9, 0.672 for SAVE-6, 0.708 for CAS, 0.700 for FCV-19S, and 0.731 for CAS-7 (Figure 2). The cutoff scores of each rating scale were set as 22 points for SAVE-9 (sensitivity = 80.2%, specificity 54.3%), 15 points for SAVE-6 (sensitivity = 76.7%, specificity 48.6%), two points for CAS (sensitivity = 54.7%, specificity = 82.3%), 19 points for FCV-19S (sensitivity = 65.1%, specificity 67.9%), and 10 points for CAS-7 (sensitivity = 66.3%, specificity = 67.9%).

## 4. Discussion

In this study, we observed that the SAVE-9, SAVE-6, CAS, FCV-19S, and CAS-7 scales showed good reliability of internal consistency among healthcare workers. Their construct validity was good, and the convergent validity of each scale was similarly good. Furthermore, when we compared the psychometric properties of the SAVE-9 scale with other rating scales, we observed that the CAS scale was the most discriminating and difficult among the scales. CAS and FCV-19S provided more information and were more efficient than the SAVE-9, SAVE-6, and CAS-7 scales when they were used to measured healthcare workers’ viral anxiety.

All viral anxiety scales showed good reliability of internal consistency and good fit for the single-factor model. They could also measure the viral anxiety of healthcare workers similarly across sex or having depression. This shows that all viral anxiety scales which were developed for measuring the anxiety response of the general population can be applied to healthcare workers in this pandemic. However, factor loadings of items 6 (Do you feel skeptical about your job after going through this experience?) and 7 (After this experience, do you think you will avoid treating patients with viral illnesses?) of the SAVE-9 scale were low among this sample. A possible explanation for the low factor loadings is that healthcare workers might adapt to long periods of the pandemic, and they might answer that they would keep working regardless of the viral outbreak.

From the scale information curve, the CAS was the most discriminating and difficult rating scale that could be applied to healthcare workers among viral anxiety rating scales in this study. However, the SAVE-9 and SAVE-6 scales were the least discriminating and difficult, and the CAS-7 and FCV-19S scales were moderate. When we conducted the ROC analysis of the viral anxiety rating scales according to the mild degree of the GAD-7 (5 point), we observed that the SAVE-9 and SAVE-6 scales were the most sensitive rating scales, and the CAS scale was the most specific rating scale. Healthcare workers suffer from severe stress while taking care of infected patients. In this context, the SAVE-9 scale was purposely developed to identify healthcare workers who need psychological support, and we defined the cut-off according to the mild degree of GAD-7 to identify healthcare workers with at least a mild degree of viral anxiety in this pandemic. Therefore, sensitivity is an important factor of the SAVE-9 scale when it is applied. The SAVE-6 was also derived from the SAVE-9 scale to assess the viral anxiety of the general population to identify individuals with mild degree of viral anxiety. In contrast, CAS was developed to screen individuals with functional impairment caused by the coronavirus pandemic [19], and it may be suitable for clinical samples. Therefore, specificity is an important factor of the CAS. Although it might depend on the purposes of applying each rating scale, SAVE-9 and SAVE-6 can be applied as screening tools for the viral anxiety of healthcare workers; in comparison, CAS can be used as a confirmatory tool.

SAVE-9 or SAVE-6, tools for screening individuals with mild degree anxiety, were placed at the other extreme from CAS, which was developed for somatic anxiety, which can be considered as a relatively severe form (somatic) of viral anxiety. However, FVC-19S and CAS-7 were moderately discriminating among a healthcare worker sample. The CAS-7 scale was developed on the basis of the symptoms of Generalized Anxiety Disorder. It includes a type of concern that is difficult to control and is persistent and excessive, along with some symptoms such as restlessness or nervousness, fatigue, difficulty concentrating, irritability, and a decreased ability to engage in social activities. The scale evaluates a single factor and consists of seven items designed to capture anxiety associated with the COVID-19 pandemic. FCV-19S was developed to measure individuals’ fear about COVID-19 based on a fear of disease. It can be clustered into “physical fear” and “emotional fear” [20]. Previously, according to the Rasch model, items of FCV-19S were reportedly distributed from low to high difficulty [18]. In addition, we can consider that the discriminating ability and difficulty of CAS-7 and FCV-19S is moderate compared to the SAVE-6 or the CAS scales when applied to healthcare workers.

This study had limitations. First, the study was conducted on 29 November 2021. From 1 November 2021, the “living with coronavirus” policy was announced by the Korean government. A rapid increase in the number of infected cases might have influenced the results. Second, participants were healthcare workers in one hospital, and the results are, therefore, not generalizable. Third, a high proportion of nursing professionals and females participated in this survey, which might limit the generalizability of the findings, in that higher levels of stress or viral anxiety among female or nursing professionals were reported [36]. Fourth, this study was conducted only in a single center. Although it was conducted in the largest hospital in South Korea, the results may be limited when applied in another center. Lastly, an anonymous online survey study might lead to bias. Furthermore, we could not discriminate against participants who were working in the frontline or taking care of infected patients.

## 5. Conclusions

We observed that viral anxiety rating scales (developed for the general population) can be applied to healthcare workers with a good reliability and validity. We also observed that each rating scale has characteristics of difficulty or is discriminatory. Therefore, we need to consider which rating scale should be applied on the basis of the characteristics of the rating scale.

## Figures and Tables

**Table 1:** Clinical characteristics of participants (*N* = 329).

Variables	*N* (%) Mean ± SD
**Sex (female)**	267 (81.4%)
Age	35.8 ± 14.3
Years of employment	9.7 ± 7.7
**Job**	
Nursing professionals	194 (59.0%)
Doctors	23 (7.0%)
Other healthcare workers	112 (34.0%)
**Marital Status**	
Single	157 (47.7%)
Married, without kids	51 (15.5%)
Married, with kids	121 (36.8%)
**Are you a shift worker? (yes)**	73 (22.3%)
**Questions on COVID-19**	
Did you experience being quarantined due to infection with COVID-19? (Yes)	45 (13.7%)
Did you experience being infected with COVID-19? (Yes)	2 (0.6%)
Did you get vaccinated? (Yes)	327 (99.4%)
**Psychiatric history**	
Did you experience or treated depression, anxiety, or insomnia? (Yes)	46 (13.9%)
At present, do you think you are depressed or anxious, or do you need help for your mood state? (Yes)	24 (7.3%)

**Table 2:** Scale level psychometric properties of viral anxiety rating scales and GAD-7 among healthcare workers (*N* = 329).

Psychometric Properties	SAVE-9	SAVE-6	CAS	FCV-19	CAS-7	GAD-7	Suggested Cutoff
**Floor effect**	0	0	59.0	3.5	4.1	29.3	15%
**Ceiling effect**	0.9	1.9	0.3	0.3	0.6	0	15%
**Mean inter-item correlation**	0.314	0.403	0.544	0.474	0.477	0.572	0.15~0.50
**Cronbach’s alpha**	0.799	0.799	0.838	0.861	0.865	0.899	≥0.7
**McDonald’s omega**	0.800	0.802	0.837	0.865	0.868	0.892	≥0.7
**Split-half reliability (odd–even)**	0.821	0.810	0.818	0.891	0.856	0.904	≥0.7
**Standard error of measurement**	2.54	1.88	0.85	2.00	1.44	1.42	<SD (5.25)/2
***Rho* coefficient**	0.800	0.798	0.861	0.866	0.882	0.897	≥0.7
**IRT reliability**	0.846	0.832	0.633	0.875	0.881	0.804	≥0.7
**Model fits of confirmatory factor analysis**							
***χ^2^* (df, *p* value)**	27.969	5.794	0.988	14.125	45.123	3.081	Nonsignificant
**CFI**	0.999	1.000	1.000	1.000	0.974	1.000	>0.95
**TLI**	0.999	1.000	1.114	1.000	0.960	1.023	>0.95
**RMSEA**	0.011	0.000	0.000	0.005	0.084	0.000	<0.08
**SRMR**	0.049	0.034	0.059	0.041	0.089	0.034	<0.08

Note: SAVE-9, Stress and Anxiety to Viral Epidemics—9 items; CAS, Coronavirus Anxiety Scale; FCV-19S, Fear of COVID-19 Scale; CAS-7, COVID-19 Anxiety Scale—7 items; GAD-7, Generalized Anxiety Scale—7 items.

**Table 3:** Corrected item-total correlation and factor loading of viral anxiety scales and GAD-7 among healthcare workers (*N* = 329).

Items	SAVE-9		SAVE-6		CAS		FCV-19S		CAS-7		GAD-7	
	CITC	FL	CITC	FL	CITC	FL	CITC	FL	CITC	FL	CITC	FL
Item 1	0.495	0.565	0.504	0.570	0.638	0.704	0.618	0.658	0.647	0.701	0.707	0.758
Item 2	0.661	0.758	0.682	0.787	0.652	0.728	0.580	0.610	0.629	0.644	0.800	0.865
Item 3	0.568	0.663	0.611	0.699	0.678	0.735	0.598	0.645	0.745	0.822	0.650	0.688
Item 4	0.558	0.656	0.604	0.686	0.647	0.727	0.591	0.646	0.695	0.767	0.772	0.828
Item 5	0.458	0.506	0.445	0.497	0.711	0.773	0.733	0.802	0.610	0.641	0.689	0.734
Item 6	0.312	0.332	0.504	0.569			0.660	0.721	0.549	0.545	0.615	0.645
Item 7	0.373	0.401					0.651	0.718	0.585	0.626	0.722	0.769
Item 8	0.545	0.611										
Item 9	0.496	0.546										

Note: CITC, corrected item–total correlation; FL, factor loading; SAVE-9, Stress and Anxiety to Viral Epidemics—9 items; CAS, Coronavirus Anxiety Scale; FCV-19S, Fear of COVID-19 Scale; CAS-7, COVID-19 Anxiety Scale—7 items; GAD-7, Generalized Anxiety Scale—7 items.

**Table 4:** Item fits, slope, and threshold parameters of viral rating scales among healthcare workers (*N* = 329).

Items	Item Fits			Slope Parameter (α)	Threshold Parameter (b)			
	S-χ^2^	df	*p*-Value		b_1_	b_2_	b_3_	b_4_
**(A) SAVE-9**								
Item 1	26.061	31	0.808	1.581	−3.345	−2.352	−1.057	0.767
Item 2	29.117	33	0.808	2.597	−2.367	−1.218	−0.244	1.352
Item 3	58.998	33	0.036	2.000	−2.855	−1.188	−0.234	1.515
Item 4	39.634	37	0.807	1.777	−2.492	−1.068	−0.369	1.346
Item 5	40.471	42	0.807	1.127	−1.207	0.558	1.691	3.034
Item 6	48.593	53	0.807	0.700	−2.784	0.155	1.727	3.776
Item 7	38.557	42	0.807	0.797	−4.781	−1.654	−0.056	3.126
Item 8	21.989	36	0.968	1.470	−3.455	−2.194	−1.065	0.904
Item 9	41.397	41	0.808	1.195	−3.852	−1.812	−0.983	0.992
**(B) SAVE-6**								
Item 1	22.124	24	0.572	1.584	−3.339	−2.345	−1.051	0.771
Item 2	21.922	20	0.572	2.778	−2.316	−1.191	−0.242	1.330
Item 3	18.369	20	0.572	2.101	−2.796	−1.163	−0.230	1.492
Item 4	26.666	26	0.572	1.827	−2.450	−1.054	−0.368	1.334
Item 5	30.776	30	0.572	1.099	−1.226	0.570	1.720	3.084
Item 6	42.030	29	0.336	1.340	−3.681	−2.319	−1.123	0.962
**(C) CAS**								
Item 1	-	0	-	2.314	0.977	2.109	2.740	3.093
Item 2	-	0	-	2.872	0.584	1.753	2.589	3.098
Item 3	-	0	-	4.158	1.574	2.303	2.826	3.332
Item 4	3.706	2	0.157	2.712	1.109	2.270	3.569	-
Item 5	4.582	1	0.064	3.786	1.217	2.361	3.390	-
**(D) FCV-19S**								
Item 1	15.162	26	0.954	1.695	−1.517	−0.407	1.053	3.045
Item 2	13.292	22	0.954	1.848	−1.982	−1.321	−0.594	1.931
Item 3	20.402	23	0.954	2.357	0.117	1.181	2.143	3.530
Item 4	39.578	31	0.954	1.745	−0.683	0.374	1.171	2.849
Item 5	18.478	23	0.954	2.828	−0.895	0.013	0.630	2.357
Item 6	18.976	21	0.954	2.628	−0.085	0.910	2.112	2.997
Item 7	42.543	28	0.273	2.157	−0.420	0.555	1.092	2.845
**(E) CAS-7**								
Item 1	16.315	13	0.408	2.393	−1.730	−0.743	1.519	
Item 2	14.246	13	0.469	2.234	−0.004	1.398	3.401	
Item 3	18.595	12	0.250	3.930	−1.111	−0.249	1.733	
Item 4	13.485	14	0.489	2.743	−1.193	−0.267	1.951	
Item 5	16.745	16	0.469	1.867	−0.369	1.016	2.636	
Item 6	18.312	12	0.250	1.826	0.357	2.092	3.430	
Item 7	35.166	16	0.028	1.999	−1.860	−0.841	1.616	
**(F) GAD-7**								
Item 1	7.351	6	0.565	3.236	0.401	1.923	3.281	
Item 2	4.719	7	0.694	4.745	0.572	1.553	2.665	
Item 3	15.837	14	0.565	2.152	−0.029	1.308	2.030	
Item 4	8.328	5	0.490	3.774	0.383	1.773	2.467	
Item 5	3.899	6	0.694	4.026	1.122	2.015	-	
Item 6	11.637	12	0.665	1.791	−0.048	1.359	2.475	
Item 7	8.307	5	0.490	3.786	0.924	1.943	3.257	

Note: SAVE-9, Stress and Anxiety to Viral Epidemics—9 items; CAS, Coronavirus Anxiety Scale; FCV-19S, Fear of COVID-19 Scale; CAS-7, COVID-19 Anxiety Scale—7 items; GAD-7, Generalized Anxiety Scale—7 items.

**Table 5:** Infit and outfit MnSQs, and item and person separation index and reliability (*N* = 329).

Scale	Items	MnSq		Difficulty	Separation Index		Reliability	
		Infit	Outfit		Item	Person	Item	Person
**SAVE-9**	Item 1	0.91	0.90	−0.85	9.984	2.047	0.990	0.807
	Item 2	0.62	0.61	−0.13				
	Item 3	0.77	0.78	−0.07				
	Item 4	0.96	0.96	−0.11				
	Item 5	1.12	1.09	1.41				
	Item 6	1.42	1.52	0.96				
	Item 7	1.10	1.12	0.13				
	Item 8	0.86	0.86	−0.75				
	Item 9	1.12	1.19	−0.59				
**SAVE-6**	Item 1	1.06	1.02	−0.93	10.856	2.086	0.992	0.813
	Item 2	0.08	0.68	−0.05				
	Item 3	0.83	0.83	0.02				
	Item 4	1.01	1.00	−0.03				
	Item 5	1.26	1.29	1.79				
	Item 6	1.10	1.16	−0.80				
**CAS**	Item 1	1.30	1.20	−0.54	4.585	0.999	0.955	0.500
	Item 2	1.04	1.04	−1.31				
	Item 3	0.90	0.53	1.29				
	Item 4	1.03	0.99	0.04				
	Item 5	0.80	0.77	0.51				
**FCV-19S**	Item 1	1.00	1.15	−0.54	12.943	2.430	0.994	0.855
	Item 2	1.05	0.95	−2.10				
	Item 3	0.87	0.78	1.40				
	Item 4	1.26	1.24	0.15				
	Item 5	0.87	0.85	−0.36				
	Item 6	0.74	0.74	1.11				
	Item 7	1.10	1.07	0.34				
**CAS-7**	Item 1	1.07	1.02	−1.94	15.090	2.485	0.996	0.861
	Item 2	0.82	0.74	1.97				
	Item 3	0.80	0.73	−0.94				
	Item 4	0.93	0.87	0.90				
	Item 5	1.10	1.14	1.19				
	Item 6	0.89	1.00	2.64				
	Item 7	1.22	1.33	−2.01				
**GAD-7**	Item 1	0.88	0.89	0.02	7.434	2.012	0.982	0.802
	Item 2	0.71	0.58	0.23				
	Item 3	1.39	1.36	−1.52				
	Item 4	0.76	0.77	−0.11				
	Item 5	0.79	0.50	1.67				
	Item 6	1.46	1.49	−1.45				
	Item 7	0.80	0.60	1.15				

Note: SAVE-9, Stress and Anxiety to Viral Epidemics—9 items; CAS, Coronavirus Anxiety Scale; FCV-19S, Fear of COVID-19 Scale; CAS-7, COVID-19 Anxiety Scale—7 items; GAD-7, Generalized Anxiety Scale—7 items.

## Data Availability

Not applicable.
